# microRNAs in Type 1 Diabetes: Roles, Pathological Mechanisms, and Therapeutic Potential

**DOI:** 10.3390/ijms26073301

**Published:** 2025-04-02

**Authors:** Hayeong Cho, Se Eun Ha, Rajan Singh, David Kim, Seungil Ro

**Affiliations:** Department of Physiology & Cell Biology, University of Nevada School of Medicine, Reno, NV 89557, USA; hayeongc5305@gmail.com (H.C.); seeunh@med.unr.edu (S.E.H.); rajans@med.unr.edu (R.S.); davidskim@med.unr.edu (D.K.)

**Keywords:** type 1 diabetes, autoimmune disease, β-cells, apoptosis, miRNAs

## Abstract

Type 1 diabetes (T1D) is a chronic autoimmune disease characterized by the progressive destruction of pancreatic β-cells, leading to insulin deficiency. The primary drivers of β-cell destruction in T1D involve autoimmune-mediated processes that trigger chronic inflammation and ultimately β-cell loss. Regulatory microRNAs (miRNAs) play a crucial role in modulating these processes by regulating gene expression through post-transcriptional suppression of target mRNAs. Dysregulated miRNAs have been implicated in T1D pathogenesis, serving as both potential diagnostic biomarkers and therapeutic targets. This review explores the role of miRNAs in T1D, highlighting their involvement in disease mechanisms across both rodent models and human patients. While current antidiabetic therapies manage T1D symptoms, they do not prevent β-cell destruction, leaving patients reliant on lifelong insulin therapy. By summarizing key miRNA expression profiles in diabetic animal models and patients, this review explores the potential of miRNA-based therapies to restore β-cell function and halt or slow the progression of the disease.

## 1. Introduction

Diabetes mellitus, commonly referred to as diabetes, is characterized by chronic hyperglycemia and associated with metabolic dysfunctions in carbohydrates, fats, and proteins [1]. The disease encompasses genetic, pathophysiological, and clinical factors [2]. Diabetes is categorized into several types based on its etiology and pathogenesis: type 1 diabetes (T1D), type 2 diabetes (T2D), gestational diabetes (GD), and other forms [1]. T1D, or insulin-dependent diabetes, results from dysfunction of pancreatic β-cells and accounts for about 5–10% of all diabetes cases [1]. T2D, or non-insulin-dependent diabetes, is primarily caused by insulin resistance and/or insulin deficiency, making up 90–95% of all cases [1]. GD is diagnosed when glucose intolerance or diabetes develops during pregnancy, typically in the second or third trimester, and affects 1–14% of pregnancies [1]. All forms of diabetes share the common feature of insulin dysregulation, either absolute or relative. Currently, no single definitive biomarker reliably assesses insulin dysregulation for diagnosing diabetes before its onset. Although in clinical research settings various tests to assess insulin secretion, such as fasting indices, oral and intravenous glucose tolerance tests, and other provocative challenges, have been proposed, they are not widely used due to their time-consuming nature, expense, and lack of standardization [3]. Furthermore, these methods do not adequately reflect the underlying pathophysiology of β-cell dysfunction, as their correlation with β-cell mass is limited [3].

**Figure 1:**
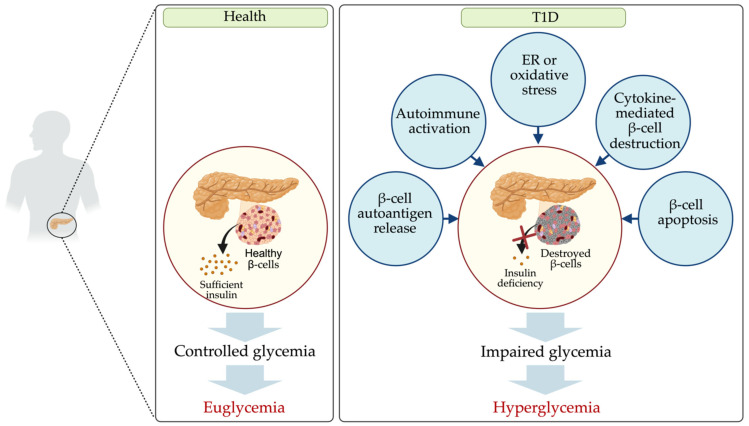
Pathophysiological mechanisms underlying Type 1 diabetes (T1D). T1D results from the progressive destruction of pancreatic islet β-cells, driven by autoantigen release, autoimmune activation, oxidative and endoplasmic reticulum (ER) stress, cytokine-induced damage, and apoptosis. This cascade of pathological events leads to β-cell loss, insulin deficiency, and hyperglycemia, ultimately disrupting insulin signaling pathways.

T1D, also known as one of the most common chronic diseases of childhood, affected approximately 8.4 million patients globally in 2021, according to the World Health Organization [4]. T1D results from the loss of pancreatic islet β-cells, often through autoimmune activation, β-cell autoantigen release, oxidative and endoplasmic reticulum (ER) stress, and cytokine-mediated damage, leading to β-cell destruction, insulin deficiency, and hyperglycemia through disruption of insulin signaling pathways (Figure 1) [5,6,7,8]. Although diverse causes of destruction of pancreatic islet β-cells were identified, the molecular mechanisms of the loss of pancreatic islet β-cells remains controversial.

There are a few biomarkers available to distinguish T1D from other types of diabetes. In autoimmune T1D, autoantibodies (AAbs) against β-cell antigens are produced early, often before clinical symptoms appear [9]. This early antibody production is often accompanied by genetic mutations associated with the disease, with variations in the human leukocyte antigen (HLA) class II genes being the most common [10]. The presence of two or more AAbs indicates a high risk for T1D, as it signals an active autoimmune response against β-cells [7,8]. Several AAbs targeting islet cells, insulin, tyrosine phosphatases (IA-2 and IA-2β), glutamic acid decarboxylase, and zinc transporter 8 serve as biomarkers for T1D [3,7,8,11,12]. While these AAbs and measurements of T-cell reactivity reliably identify individuals at risk of T1D by assessing β-cell dysfunction due to autoimmune activity, they lack precision in predicting T1D onset and are not effective for monitoring disease progression [3,11,12].

Furthermore, while AAbs are the most common biomarkers for T1D, they are only applicable for identifying T1D in AAb-positive individuals. Notably, some children in the initial stage may be negative for islet AAbs and some AAb-positive individuals may not develop T1D [7,8]. Moreover, despite extensive research into the molecular mechanisms underlying T1D pathogenesis, patients still rely on lifelong insulin therapy, which has limited tolerance and potential adverse effects [13]. Thus, novel biomarkers to assess β-cell dysfunction are essential to predict T1D onset, monitor its progression, and establish effective clinical approaches in T1D therapies.

microRNAs (miRNAs) can serve as biomarkers and therapeutic targets in various human diseases [14]. miRNAs are small, non-coding RNA molecules, typically 22 nucleotides in length, that regulate gene expression [15]. They do so by binding to the 3′ untranslated regions of target messenger RNAs (mRNAs), forming the RNA-induced silencing complex (RISC) [8,16]. This RISC complex directly suppresses the translation of mRNAs into proteins [8,16]. An increasing number of studies suggest miRNAs hold significant potential as biomarkers for pathogenic conditions, and as therapeutic agents for medical intervention in nearly all human disease, including T1D and T2D. Studies have shown that miRNA dysregulation are associated with T1D and hyperglycemia pathogenesis, highlighting their potential as biomarkers for T1D. miR-125b-5p and miR-365a-3p showed positive correlation with hemoglobin A1C levels [17], whereas let-7a-5p, let-7c-5p, miR-5190, and miR-770-5p exhibited negative correlations [17,18]. These miRNAs significantly influence glycosaminoglycan biosynthesis, axon guidance signaling, Rap1 signaling, focal adhesion, and neurotrophin signaling, linking these pathways to T1D pathology [17,19].

Recent studies have emphasized the therapeutic potential of miRNA-based strategies, such as using miRNA mimics to restore downregulated miRNAs or employing miRNA inhibitors to counteract overexpressed miRNAs, thereby achieving protein homeostasis [20,21]. The safety of prolonged treatment with specific miRNAs has also been demonstrated in preclinical models [22]. With six FDA-approved siRNA drugs (Patisiran, Givosiran, Lumasiran, Inclisiran, Vutrisiran, and Nedosiran) now in clinical use for genetic and metabolic disorders, ongoing research on miRNAs—mimicked by these siRNAs—continues to expand their applications in both preclinical and clinical settings. However, several critical challenges remain to fully harness the potential of miRNA-based therapies. These include identifying key signature miRNAs, elucidating their mechanisms of action, optimizing their use through RNAi, ensuring efficient delivery to target tissues, and validating their efficacy in vivo. Although several miRNA-based therapeutics have entered clinical trials [14], none have yet achieved FDA approval.

Numerous studies have identified dysregulated miRNAs in both T1D rodent models and patients. This review explores differentially expressed miRNAs in T1D, emphasizing their interactions with target genes involved in T1D-related pathways to uncover the molecular mechanisms underlying β-cell dysfunction in rodents and humans. Furthermore, it highlights commonly upregulated and downregulated miRNAs shared between T1D rodent models and patients. These insights aim to advance the development of diagnostic and therapeutic strategies for T1D.

## 2. Dysregulated miRNAs in T1D Patients

Tissue-specific genes regulate cell phenotype and function, shaping distinct tissue development [23,24]. miRNAs, which exhibit both tissue- and developmental-stage-specific features, play an essential role in tissue identity and function [24,25,26]. Numerous studies have demonstrated correlations between tissue-specific miRNAs and various human diseases [24]. Circulating miRNAs, influenced by tissues such as the heart, liver, pancreas, kidney, colon, and lung, have also been shown to play key roles in disease processes, with many originating from blood cells [27]. These circulating miRNAs are easily accessible, relatively stable, and exhibit disease-specific profiles [28].

### 2.1. Dysregulated miRNAs in Various Samples from T1D Patients

Over the past decades, numerous studies have documented the dysregulation of miRNAs in various specimens, including serum, plasma, peripheral blood, peripheral blood mononuclear cells (PBMC), and exosomes from T1D patients. Table 1 provides a summary of dysregulated miRNAs identified across these sample types. In T1D patients, miRNAs exhibit differential upregulation and downregulation depending on the sample type, with certain miRNAs consistently dysregulated across multiple specimens.

**Table 1:** Altered miRNA profiles in blood from T1D patients.

Sample	Expression	miRNA	Fold Change (vs HC *)	Ref.
Serum	Up	miR-21-5p miR-24-3p miR-25 miR-27a miR-29a miR-140-5p miR-144-5p miR-148a miR-152 miR-181a miR-199a miR-200a miR-208a-3p miR-210 miR-222-3p miR-323a-3p miR-345-5p miR-454-3p	NA ** 1.6–2.25 1.53 2.79 2.39 1.7 5.1 2.25 2.09 2.30 NA 1.23–2.73 1.97 1.65 2.7 1.8–5.6 2.3 2.6	[29] [18,30] [30] [30] [30] [18] [18] [29,30] [30] [30] [30] [30,31] [31] [30] [18] [31] [18] [18]
	Down	miR-16-5p miR-19a-3p miR-25-3p miR-155-5p ^2^ miR-195-5p miR-590-5p	0.38 0.42 0.40 0.26-0.54 0.42 0.46	[31] [31] [31] [31,32] [31] [31]
Plasma	Up	miR-10b-5p ^1^ miR-21 miR-24 miR-103a-3p miR-125b-5p miR-146a-5p ^2^ miR-155-5p ^1^ miR-200a-3p miR-210-3p miR-365a-3p miR-770-5p	NA NA NA 121.0 NA NA 1.88 59.90 4.79 NA NA	[33] [34] [3] [32] [17] [3,32] [32] [32] [32,34] [17] [17]
	Down	miR-10b-5p ^2^ miR-146a-5p ^1^ miR-5190 miR-409-3p	NA 0.29 NA NA	[33] [32] [17] [35]
Peripheral blood cells	Up	miR-17-5p miR-21-5p miR-26a-5p miR-181a-5p miR-221-3p	NA NA NA NA NA	[36] [36] [36] [36] [36]
	Down	miR-16-5p miR-17-5p miR-126-3p/5p miR-143-3p	NA NA NA NA	[36] [36] [36] [36]
PBMC ^3^	Up	miR-21 miR-22 miR-26b miR-32 miR-92-3p miR-126 miR-142-5p miR-143-3p miR-148a/b miR-186 miR-195	3.46 NA 2.21 2.11 NA 3.36 NA NA 1.60–2.01 1.65 2.16	[19] [37] [19] [19] [38] [19] [38] [38] [19] [19] [19]
	Down	miR-140-3p miR-146a/b miR-150 miR-423-5p miR-424 miR-720	0.63 NA NA 0.55 NA 0.56	[19] [39,40] [37,38,40] [19] [40] [19]
Plasma-derived exosome	Up	miR-25-3p	NA	[41]
	Down	miR-16 miR-302d-3p miR-378e	NA NA NA	[41] [41] [41]

^1^ T1D diagnosed within 5 years, ^2^ T1D after 5 years, ^3^ PBMC, peripheral blood mononuclear cells, * HC, healthy control; ** NA, data not available.

Recent research has identified 44 upregulated and 22 downregulated miRNAs that are consistently altered in T1D patients compared to healthy donors (Table 1). Among them, five miRNAs in various specimens were reported to be dysregulated in T1D patients: miR-21 [3,19,29,30,31,32,34] and miR-148a [3,19,29,30,31,32] were upregulated, while miR-126 [31,32,34,36] was downregulated. miR-25-3p [18,32,36,41] and miR-1275 [19,32,39] exhibited distinct dysregulation patterns in various specimens. miR-21 and miR-148a regulate inflammation and apoptosis. In patients with T1D, the increased expression of miR-21 and miR-148a regulates PI3K/AKT signaling and contributes to apoptosis in β-cells leading to dysregulated insulin release through impaired glucose-stimulated insulin secretion through PTEN and SOX6 [29,42,43,44]. Conversely, the decreased expression of miR-25-3p, miR-126, and miR-1275 targets IL-1β and IRS-1, promoting insulin-producing β-cell death [45,46,47,48,49].

Notably, one interesting study [32] found that both miR-148a-3p and miR-148b-3p levels in plasma were increased in newly diagnosed T1D patients (<5 years of diagnosis) but decreased in later-stage T1D patients (≥5 years of diagnosis). These miRNAs are involved in the regulation of the Wnt, FOXO, and insulin signaling pathway [29,32]. In the early stages of T1D, pancreatic β-cell damage progresses due to autoimmunity [5]. During this stage, the increased expression of miR-148a/b-3p regulates the Wnt and insulin signaling pathways, protecting the remaining pancreatic β-cells or modulating inflammatory responses. In later stages, as pancreatic β-cell damage worsens, pancreatic function is lost, and chronic inflammation leads to fibrosis [1,5], the role of miR-148a/b-3p diminishes, and its expression levels decrease. These findings suggest that miR-148a/b-3p may serve as valuable biomarkers distinguishing different stages of T1D.

### 2.2. Dysregulated miRNAs in Serum, Plasma, and Exosomes Derived from Plasma

Circulating miRNAs, found in nearly all biological fluids such as serum and plasma, represent promising sensitive biomarkers for a range of conditions, as their profiles provide an accurate representation of the physiological state of the organism [50,51,52]. In particular, miRNAs expressed in serum exhibit changes in expression levels in response to disease states and physiological changes [52,53,54,55]. They exhibit altered expression profiles in various pathological conditions, including metabolic diseases such as diabetes and inflammatory disorders [50,51,55,56,57,58]. These changes make them valuable biomarkers for early disease diagnosis, prognosis evaluation, and monitoring therapeutic responses.

Research into the expression of these miRNA markers suggests their potential application in the early diagnosis of various diseases, frequently preceding the manifestation of clinical symptoms [54,59,60,61,62,63]. Additionally, these markers may play a crucial role in evaluating patient responses to therapeutic treatments, thus aiding in formulating personalized treatment approaches [64,65].

The analysis of miRNA profiles in T1D patients highlights distinct patterns of dysregulation in both serum and plasma, underscoring their potential as biomarkers for the disease. Specifically, in T1D, 18 miRNAs were upregulated and 6 were downregulated in serum, while 11 miRNAs were upregulated and 4 were downregulated in plasma (Table 1). Notable examples include the downregulation of miR-10b-5p, miR-146a-5p, and miR-409-3p [18,29,66] and the upregulation of miR-21, miR-126, and miR-155-5p [17,67,68,69], which are associated with key pathways in T1D pathogenesis.

Exosomes, secreted by all cell types [70], are lipid-based carriers with the potential for disease-specific diagnostics, since they originate from cellular multivesicular bodies that contain specific miRNAs [41,71]. Exosomal miRNAs are tissue-specific and remain stable in plasma, whereas unprotected RNAs including miRNAs in blood are prone to degradation by ribonucleases [41,71,72]. miRNA expression patterns dynamically change throughout disease progression. Pancreatic β-cells release exosomes containing miRNAs [73]. miRNAs derived from exosomes in plasma are promising biomarker candidates for T1D. Dysregulated exosomal miRNAs play a critical role in T1D pathophysiology, influencing β-cell function [30,41,74], insulin resistance [41,75], and metabolic regulation [41]. Notably, exosomal miR-302d-3p, miR-378e, and miR-16 are downregulated, while miR-25-3p is upregulated (Table 1).

Moreover, miRNA expression levels change dynamically with T1D progression; for instance, miR-10b-5p and miR-155-5p are upregulated in T1D patients diagnosed within the first 5 years but becomes downregulated thereafter, while miR-146a-5p exhibits the opposite pattern, being initially downregulated and later upregulated [32,33] (Table 1). These findings suggest that miRNAs can provide insights into T1D mechanisms and can serve as a foundation for developing diagnostic and therapeutic tools for T1D progression.

### 2.3. Dysregulated miRNAs in Blood Cells

Approximately 80% of all human genes are expressed in peripheral blood cells, including PBMC [76]. These cells are highly responsive to environmental changes, which can significantly impact their gene expression profiles [19,76]. In the context of T1D, hundreds of miRNAs have been reported to exhibit differential expression in PBMC [8]. The dysfunction of PBMC, particularly T-cells and B-cells, plays a critical role in the pathogenesis of autoimmune diseases such as T1D [5]. The distinct miRNA expression patterns in PBMC hold promise as valuable biomarkers for the diagnosis and monitoring of T1D.

The evidence presented highlights the critical role of dysregulated miRNAs in the pathogenesis and progression of T1D. Studies by Takahashi et al. [19], Yang et al. [39], and Massaro et al. [38] collectively demonstrate that specific miRNA expression patterns can distinguish T1D patients, implicating these molecules in key pathways such as immune regulation, insulin signaling, and diabetic complications. Notably, miR-146a/b-5p emerge as central players in the autoimmune imbalance underlying the onset of T1D, with their persistent downregulation linked to immune dysregulation and T-cell response (Table 1). Furthermore, the target genes of miR-146a/b-5p, such as TRAF6, BCL11A, STX3, and NUMB, were upregulated in PBMC from newly diagnosed T1D patients and those treated with insulin [39]. These target genes are involved in immune regulation and T-cell responses. For instance, TRAF6 regulates TLR signaling to maintain immunological balance [39,67] and NUMB inhibits the Notch signaling pathway, which plays a critical role in T helper cell immune responses [39,77]. STX3, involved in chemokine production by human mast cells [39,78], and BCL11A, related to plasmacytoid dendritic cells differentiation, are also key immune-related genes [39,79]. Additionally, distinct miRNA signatures associated with complications such as neuropathy, nephropathy, and retinopathy further underscore the potential of miRNAs as biomarkers for disease progression and therapeutic targets [49,80,81,82,83,84,85,86]. These findings pave the way for future research into miRNA-targeted therapies aimed at mitigating both the autoimmune aspects of T1D and its associated complications.

### 2.4. Dysregulated miRNAs in T-Cells

T1D is characterized by the loss of functional insulin-producing β-cells in the pancreatic islets due to their immune-mediated destruction. This phenomenon is caused by islet infiltrating self-reactive CD4^+^ and CD8^+^ T-cells [87]. Simultaneously, regulatory T-cells (Tregs) suppress these autoreactive T-cells [88]. Although most self-reactive T-cells are eliminated in the thymus via the central tolerance induction mechanism, which strengthens immune tolerance to self-antigens under normal condition, some autoreactive T-cells evade this mechanism and enter peripheral circulation [88]. This phenomenon suggests that impaired T-cell and Treg function contribute to the onset of autoimmune T1D by reducing tolerance to islet antigens, leading to uncontrolled T-cell-mediated destruction of pancreatic β-cells [88,89].

miR-191, miR-342, and miR-510 were dysregulated in Tregs isolated from peripheral blood of T1D patients [89]. Specifically, miR-191 was downregulated in Tregs of T1D patients [89,90,91,92]. miR-342, abundantly expressed in Tregs of healthy donors, was also significantly downregulated in T1D patients [89]. Furthermore, this miRNA was shown to be downregulated in human leukocytes during lipopolysaccharide-induced inflammation [89,93]. miR-342 targets key molecules such as BMPR2 and PDGFRA in cytokine signaling and EP300 in MAPK and NF-кB signaling pathways [89,93]. These observations suggest that miR-342 downregulation in Tregs of T1D patients may impair their functionality, contributing to autoimmune β-cell destruction in T1D [89,90,91,92].

## 3. Dysregulated miRNAs in T1D Rodents

As previously noted, tissue-specific miRNAs play a critical role in normal tissue development [94] and T1D progression [95]. Table 2 summarizes dysregulated miRNAs in T1D murine models across various studies. These miRNAs are categorized by diabetic models, sample types, and expression patterns, offering valuable insights into their roles in the T1D pancreas.

**Table 2:** Altered miRNA profiles across pancreas tissues and cells in T1D mouse models.

Diabetic Animal Model	Sample	miRNA Expression Alteration		Ref.
		Expression	miRNA	
Prediabetic NOD * mice	Pancreatic islet, cultured islet, infiltrating lymphocytes	Up	miR-29a miR-142-5p	[96,97] [96,97]
		Down	miR-142-3p miR-150	[96,97] [96,97]
	Pancreatic β-cells	Up	miR-142-5p	[97]
		Down	miR-150 miR-155	[97] [97]
Diabetic NOD mice	Pancreatic islet/plasma	Up	miR-21	[98]
		Down	miR-126a-3p/5p miR-155 miR-409-3p	[35] [35] [35]
C57BL/6J mice induced with STZ *	Pancreatic tissue	Up	miR-323-3p	[99]
		Down	miR-10b-5p miR-16-5p miR-17-5p miR-126-3p/5p miR-143-3p	[99] [99] [99] [99] [99]
	Pancreatic islet	Up	miR-21	[98]

* NOD, non-obese diabetic; STZ, Streptozotocin.

Various miRNAs have been implicated in T1D pathogenesis within pancreatic islet-related samples, including pancreatic islets, pancreatic β-cells, the whole pancreas, cultured islets, infiltrating lymphocytes, serum, and blood from T1D rodent models. Among these, 5 miRNAs were upregulated [35,96,97,98,99], while 10 were downregulated [35,96,97,98,99] (Table 2). Notably, miR-21 and miR-142-5p are consistently upregulated in the pancreas of both NOD and STZ diabetic models.

Among the dysregulated miRNAs implicated in T1D pathogenesis, specific miRNAs exhibit more direct roles in disease mechanisms. For example, the miR-29 family affects insulin biosynthesis in prediabetic NOD mice, while miR-142-3p, miR-142-5p, and miR-155 are linked to apoptosis and inflammation [96,97]. In addition, miR-21, which is upregulated in β-cells of both NOD and STZ-induced T1D mice (Table 2), contributes to islet inflammation and β-cell dysfunction [98]. Other notable findings include the downregulation of miR-409-3p in pancreatic islets and plasma from NOD mice (Table 2) [35], further emphasizing the role of dysregulated miRNAs in β-cell dysfunction and apoptosis.

These findings underscore the fundamental role of dysregulated miRNAs in T1D pathogenesis, driving β-cell dysfunction and loss. The identification of tissue-specific and circulating miRNAs provides insights into the molecular mechanisms of T1D and highlights their potential as biomarkers for both disease progression and therapeutic intervention.

## 4. Dysregulated miRNAs in T1D and Their Potential Targets

miRNAs play a vital role in maintaining glucose homeostasis by regulating β-cell development and survival [100]. T1D results from the progressive loss of pancreatic islet β-cells, driven by autoimmune activation, β-cell autoantigen release, oxidative and ER stress, and cytokine-mediated damage [101], ultimately leading to β-cell destruction (Figure 1). This loss disrupts insulin signaling pathways, causing insulin deficiency and hyperglycemia. Table 3 summarizes dysregulated miRNAs and their associated pathways in T1D from both human and murine studies.

**Table 3:** Altered miRNAs and their associated pathways in T1D.

Group	Pathway	Upregulated miRNA	Downregulated miRNA
Apoptosis	β-cell apoptosis	miR-21-5p [102,103] miR-24 [104] miR-34a [105] miR-155 [106] miR-181a-5p [8] miR-200a-3p [32,107] miR-375 [108] miR-424 [19]	miR-100-5p [89] miR-126 [109] miR-146a-5p [32,40] miR-150-5p [40] miR-320a-3p [110] miR-424 [40]
	TP53 signaling	miR-145 [111]	miR-324-5p [19,99] miR-342-3p [19,89] miR-423-5p [19]
	Wnt signaling	miR-143-3p [112] miR-144-5p [18] miR-148a-3p [29,32] miR-365a-3p [17]	miR-140-3p [19] miR-766 [19] miR-940 [19]
	TGF-β or mTOR signaling	miR-26b [32,113] miR-323-3p [99] miR-382-5p [38]	miR-10b-5p [114]
ER or oxidative stress	FOXO or Notch signaling	miR-21-5p [29,115] miR-148a-3p [32] miR-323-3p [99] miR-486-5p [31,116]	miR-140-3p [19] miR-146a-5p [32] miR-324-5p [19] miR-423-5p [19]
	NF-κβ signaling	miR-24-3p [117,118] miR-155-5p [32,119,120,121]	miR-146a-5p [32] miR-342 [122]
Immune system activation	Immune system-related	miR-103a-3p [32] miR-200a-3p [32]	
	T-cell regulation		miR-31 [89] miR-342 [89]
	Chemokine signaling [19]	miR-18b miR-20b miR-101 miR-186	miR-940
β-cell autoantigen release	Jak-STAT signaling	miR-21-5p [8] miR-24-3p [8,95] miR-125b-5p [17,123] miR-181-5p [8] miR-323-3p [99] miR-210-5p [8]	
	MAPK signaling	miR-199a [19] miR-342 [32] miR-450a [19] miR-548c-3p [19]	miR-100-5p [8] miR-150-5p [8]
β-cell insulin release	Insulin signaling	miR-21 [19] miR-26b [36] miR-32 [19] miR-103a-3p [32] miR-143-3p [36] miR-148a [19] miR-200a-3p [32] miR-210-3p [32] miR-320c [124] miR-424 [19] miR-1225-5p [124]	miR-29a [125] miR-146a-5p [32] miR-324-5p [19] miR-342-3p [19] miR-423-5p [19]

### 4.1. β-Cell Autoantigen Release

In T1D pathogenesis, the release of β-cell autoantigens such as insulin, GAD65, IA-2A, and ZnT8 triggers and sustains the autoimmune responses [126,127]. Inflammatory stress on β-cells activate autoreactive T cells and stimulates B cells to produce antibodies targeting β-cell antigens, contributing β-cell destruction [101,128]. Activated CD4^+^ and CD8^+^ T cells infiltrate the islets, releasing inflammatory cytokines and directly targeting β-cells, ultimately leading to insulin deficiency and hyperglycemia [129].

Research has shown that altered levels of miR-424-5p, miR-150-5p, and miR-342-3p in serum are negatively associated with T1D progression in individuals positive for islet autoantibodies. Among these, miR-424-5p exhibit the strongest correlation with disease progression, highlighting their potential role in the development of islet autoimmunity in T1D [130].

### 4.2. Autoimmune Activation

In T1D, autoimmune activation is a key driver of disease onset [131]. Normally, the immune system distinguishes between self and non-self, protecting the body from harmful pathogens [132]. However, in T1D, this system mistakenly targets insulin-producing β-cells in the pancreas [131,133]. Islet-invading T-cells attack and destroy β-cells, progressively reducing insulin production [131,134]. This immune activation is influenced by genetic predisposition and environmental factors, such as viral infections [5]. Once initiated, the autoimmune cascade leads to sustained β-cell destruction, ultimately resulting in insulin deficiency [131]. As insulin levels decline, blood glucose rises, leading to the hallmark symptoms and complications of T1D.

At a molecular level, several miRNAs have been implicated in this autoimmune process. In recently diagnosed patients (<5 years), miR-21-5p, miR-103a-3p, miR-148b-3p, miR-155-5p, and miR-210-3p are upregulated in plasma, while miR-146a-5p is downregulated [32]. These expression patterns appear specific to the early disease stages, as no significant differences are observed beyond five years post-diagnosis [32]. Among these, miR-155-5p is notably increased in activated T and B-cells, macrophages, and dendritic cells through the NF-κB and JNK pathways, underscoring its critical role in both innate and adaptive immunity [32,119,120,121]. Upregulated miR-155-5p also modulates immune responses by reducing NF-κB activation via the downregulation of its kinase, IKK downregulation [32,119,121]. In T1D, this regulatory function contributes to the autoimmune destruction of β-cells [32].

Additionally, miR-200 promotes a pro-apoptotic genetic signature in pancreatic islets of diabetic mice by increasing TP53 expression, which suppresses anti-apoptotic and stress-resistance pathways in β-cells [32,135]. miR-210-3p, upregulated in the plasma, serum, and urine of pediatric T1D patients, downregulates FOXP3, a key regulator of Treg function, impairing immune tolerance and exacerbating autoimmunity [32,136]. Due to their role in immune dysregulation, miR-21, miR-126, and miR-210 play crucial roles in the pathophysiology of diabetes [29]. In T1D patients, miR-21 and miR-210 levels are significantly elevated in both plasma and urine, whereas miR-126 is reduced in urine but remains unchanged in plasma [34].

Furthermore, research has shown that miRNA expression patterns in T1D change with disease progression, influencing immunological processes in the early stages. For example, miR-10b-5p, miR-17-5p, miR-30e-5p, miR-93-5p, miR-99a-5p, miR-125b-5p, miR-423-3p, and miR-497-5p exhibit significant temporal alterations based on disease duration [33]. Two distinct expression patterns were identified [33]. The first group—miR-17-5p, miR-30e-5p, miR-93-5p, and miR-423-3p—showed reduced expression within the first 12 months post-diagnosis but increased between one and five years. The second group—miR-10b-5p, miR-99a-5p, miR-125b-5p, and miR-497-5p—displayed elevated expression in the first 12 months, followed by a decline over the subsequent five years. Additionally, miR-30e-5p, miR-93-5p, and miR-423-3p maintained consistently higher expression levels throughout the study, whereas miR-10b-5p, miR-17-5p, miR-99a-5p, miR-125b-5p, and miR-497-5p exhibited persistently lower expression relative to the overall sample average at all time points [33]. These findings underscore the dynamic regulation of miRNAs in T1D and their potential role driving disease progression.

Guay et al. [97] demonstrated that rodent and human T lymphocytes release exosomes containing miR-142-3p/5p and miR-155, which can be transferred to pancreatic β-cells. Suppression of these miRNAs in recipient β-cells blocked exosome-mediated apoptosis and prevented diabetes development in NOD mice, leading to improved insulin levels, reduced insulitis scores, and diminished inflammation. Additionally, exosomes from T lymphocytes induced apoptosis and upregulated Ccl2, Ccl7, and Cxcl10 expression, activating chemokine signaling specifically in β-cells. This process recruited immune cells, potentially exacerbating β-cell destruction during the autoimmune attack [97].

Ventriglia et al. [35] demonstrated that miR-409-3p serves as a circulating biomarker of islet inflammation and T1D severity. This miRNA was downregulated in the immune infiltrates of diabetic NOD mice, with its pancreatic expression correlating with insulitis severity and CD8^+^ central memory T cells [35]. Additionally, plasma miR-409-3p levels progressively declined during diabetes progression and increased with disease remission following anti-CD3 antibody therapy [35], which is known to modulate the islet immune response and slow disease progression [137,138]. Similarly, decreased plasma miR-409-3p levels were observed in recently diagnosed T1D patients, showing an inverse correlation with HbA1c levels [35]. These findings suggest that circulating miR-409-3p may be a clinically applicable biomarker for T1D progression and severity.

Autoimmune activation in T1D is a complex, multifactorial process driven by genetic predisposition and environmental triggers. The key miRNAs discussed above play pivotal roles in the autoimmune destruction of β-cells, underscoring their potential as biomarkers and therapeutic targets for disease management and progression.

### 4.3. Endoplasmic Reticulum and Oxidative Stress

In T1D, endoplasmic reticulum (ER) and oxidative stress are key contributors to the progressive destruction of insulin-producing β-cells [103]. Due to their high insulin production demands and exposure to an inflammatory environment caused by autoimmune attacks, β-cells are particularly vulnerable to these stressors [139]. Oxidative stress in T1D arises from an imbalance between reactive oxygen species (ROS) production and the cell’s antioxidant defense mechanisms [140]. Upon lymphocyte infiltration into the pancreatic islets, ROS trigger the production of proinflammatory cytokines (IFN-γ, TNF-α, and IL-1) through redox-dependent signaling pathways, thereby promoting β-cell destruction [141]. The transcription factors FOXO and NRF2 play crucial roles in the antioxidant response, but their reduced activity in T1D exacerbates oxidative stress [142].

Several miRNAs regulate ER and oxidative stress mechanisms in T1D. miR-200a-3p targets KEAP1, a negative regulator of NRF2, and its downregulation in T1D impairs NRF2 activation, increasing β-cell susceptibility to oxidative damage [143]. miR-146a modulates inflammatory and stress responses by targeting TRAF6 and IRAK1, key components of the NF-κB signaling pathway [144,145]. Its dysregulation in T1D leads to increased NF-κB activity, exacerbating both ER and oxidative stress and promoting β-cell apoptosis [144]. Additionally, miR-21-5p induces oxidative stress by targeting KRIT1, NRF2, and SOD2, regulating intracellular ROS homeostasis [102,103]. Its dysregulation in T1D is associated with increased oxidative damage and impaired β-cell function [103,146].

These miRNAs and their targets form critical regulatory networks that modulate ER and oxidative stress responses in β-cells. Disruptions in these pathways accelerate β-cell destruction and T1D progression.

### 4.4. Apoptosis

Apoptosis, a tightly regulated process of programmed cell death, is essential for maintaining cellular homeostasis by eliminating compromised cells [147]. However, in T1D, excessive apoptosis of β-cells is a key driver of disease progression [148]. Cytotoxic T-cells, which typically target pathogens, mistakenly attack β-cells, releasing cytotoxic molecules such as granzyme B and perforin that trigger apoptosis [148].

Several molecular pathways mediate β-cell apoptosis in T1D. Anti-apoptotic proteins like BCL2 and MCL-1 help preserve mitochondrial integrity under normal conditions, but the proinflammatory environment of T1D—dominated by cytokines such as IL-1β, TNF-α, and IFN-γ—disrupts the balance between pro- and anti-apoptotic signals, tipping the scale toward cell death [149,150]. This imbalance promotes mitochondrial dysfunction, cytochrome c release, and caspase activation, culminating in β-cell apoptosis [148,149,150].

miRNAs play a crucial role in regulating apoptotic pathways in T1D. miR-15a-5p, miR-16-5p, miR-21-5p, miR-30e-5p, miR-34a, and miR-146a target genes involved in apoptosis [8,86,151,152,153], while miR-100-5p and miR-150-5p impact the PI3K/Akt pathway, which regulates β-cell survival and growth [8]. Chronic inflammation and oxidative stress impair PI3K/Akt signaling, weakening its protective effects on β-cells and increasing their vulnerability to apoptosis [154]. The cyclin-dependent kinase inhibitor P21, which regulates cell cycle progression, can also induce apoptosis under inflammatory conditions [155]. miR-21-5p, miR-100-5p, and miR-375 modulate the Cyclin–CDK complex, influencing β-cell proliferation and survival [8]. Additionally, KLF11 and the TGF-β pathway contribute to β-cell apoptosis, with miRNAs such as miR-10b-5p, miR-21-5p, and miR-424 modulating these pathways [156,157,158,159].

Certain miRNAs are notably upregulated in T1D. For example, miR-24, which is involved in inflammation and TGF-β signaling, is implicated in both T1D and T2D pathogenesis [30,160,161,162]. miR-25, associated with apoptosis regulation, is elevated in the serum of T1D children and negatively correlated with β-cell function [30,163,164,165,166,167,168]. Similarly, miR-21-5p and miR-148a, which are elevated in T1D patients, contribute to apoptosis through pathways such as FOXO and TGF-β, with miR-148a specifically targeting BCL2L11 to promote β-cell death [29,169]

miRNAs in T1D also exhibit complex, context-dependent roles. For example, miR-200b promotes apoptosis by downregulating Oxr1 under oxidative stress, whereas its inhibition confers protection against apoptosis [81]. The miR-29 family reduces glucose-stimulated insulin secretion and proinsulin mRNA levels but paradoxically mitigates cytokine-induced apoptosis in β-cells [96]. Similarly, miR-21-5p, upregulated in early T1D, promotes apoptosis by suppressing BCL2 [98]. Collectively, these findings underscore the intricate role of miRNAs in regulating molecular pathways that drive β-cell apoptosis in T1D pathophysiology.

### 4.5. Insulin Signaling

The pathogenesis of diabetes is closely linked to impaired insulin secretion, with both T1D and T2D involving disruptions in insulin signaling [170,171]. Numerous studies have elucidated the molecular mechanisms underlying these impairments, highlighting the roles of insulin and the insulin receptor in disease progression [172].

miR-103a-3p is upregulated in both T1D and T2D patients [32,68], where it plays a critical role in insulin signaling by targeting CAV1, a key regulator of the insulin receptor in both forms of diabetes [69]. Similarly, Ferraz et al. [36] identified 41 dysregulated miRNAs in T1D patients, with miR-21-5p having the highest number of target genes associated with insulin resistance, apoptosis, and diabetic cardiomyopathy, making it the most significant among the dysregulated miRNAs. miR-21-5p targets regulators including SOCS and AKT, which are involved in insulin signaling and apoptosis pathways [8,32,36]. This implicates that miR-21-5p contributes to nuclear and mitochondrial dysfunctions, ultimately exacerbating T1D progression [36].

**Figure 2:**
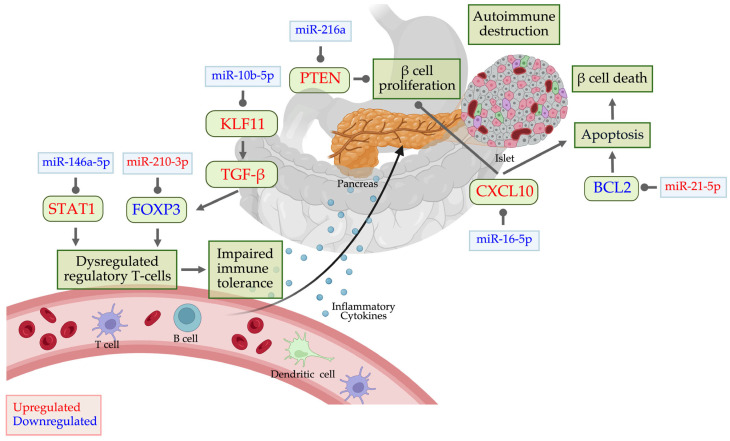
miRNA regulation in T1D pathogenesis and their target pathways. Dysregulated miRNAs play a pivotal role in T1D pathogenesis by modulating key immune and apoptotic processes. Upregulated miRNAs and their target genes are highlighted in red, while downregulated ones are shown in blue. These dysregulated miRNAs contribute to regulatory T-cell dysfunction, impaired immune tolerance, inhibition of β-cell proliferation, and apoptosis, ultimately driving autoimmune β-cell destruction.

Figure 2 illustrates dysregulated miRNAs in T1D that target key proteins, contributing to regulatory T-cell dysfunction, impaired immune tolerance, reduced β-cell proliferation, and heightened apoptosis—ultimately driving autoimmune β-cell destruction. miR-146a-5p, downregulated in T1D [173], normally inhibits STAT1 [174]. Its reduction activates STAT1 in regulatory T-cells, impairing immune tolerance [174]. miR-10b-5p, downregulated in late T1D [33], increases KLF11 expression [156], enhancing TGF-β signaling [175]. However, impaired TGF-β signaling in T1D disrupts regulatory T-cell function [176]. miR-210-3p, upregulated in T1D [32], suppresses FOXP3 [136], further contributing to immune dysregulation. miR-216a, crucial for pancreatic β-cells [177], is downregulated in T1D [178]. This reduction increases PTEN, inhibiting β-cell proliferation [178]. miR-21-5p, upregulated in T1D [8,151], suppresses the anti-apoptotic protein BCL2, leading to increased β-cell apoptosis [98]. miR-16-5p enhances β-cell proliferation by inhibiting apoptosis [152]. However, its downregulation in T1D upregulates CXCL10, promoting β-cell death [152]. Collectively, these dysregulated miRNA pathways drive β-cell destruction in T1D.

## 5. miRNA-Based Therapeutic Strategies for T1D

miRNA-based therapeutics represent an innovative approach to disease treatment by regulating gene expression at the post-transcriptional level [14]. Given that miRNA expression is altered in various diseases, modulating their levels—either by introducing miRNAs or inhibiting their function—offers a promising therapeutic strategy [179]. This concept parallels antisense mRNA and RNA interference (RNAi) techniques [179]. miRNA-based therapeutics primarily follow two strategies: antisense inhibition of mature miRNAs and miRNA replacement [14]. The choice of approach depends on whether the therapeutic goal is to suppress overexpressed miRNAs or restore downregulated miRNAs to regain lost function [14].

**Figure 3:**
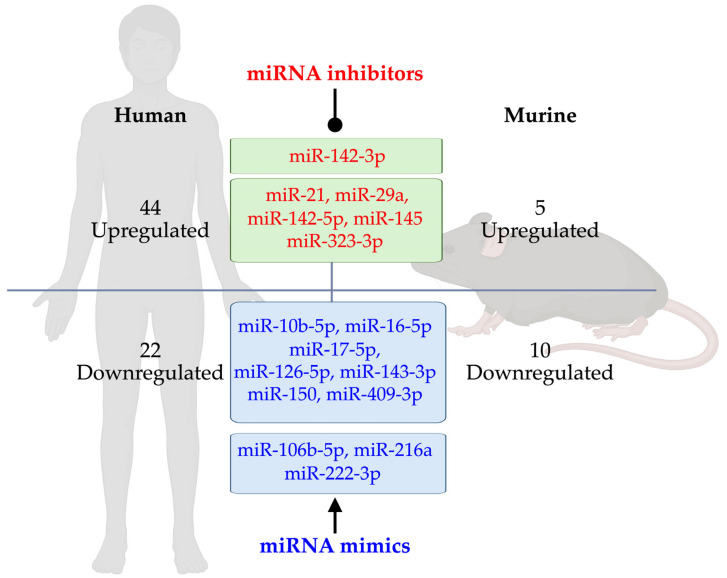
Conserved dysregulated miRNAs in T1D patients and murine models and therapeutic strategies. In T1D patients, 44 miRNAs are upregulated and 22 downregulated, while T1D animal models exhibit 5 upregulated and 10 downregulated miRNAs. Six miRNAs (miR-21, miR-29a, miR-142-3p/5p, miR-145, and miR-323-3p) are consistently upregulated in both humans and animal models. Conversely, 10 miRNAs (miR-10b-5p, miR-16-5p, miR-17-5p, miR-106b, miR-126-5p, miR-143-3p, miR-150, miR-216a, miR-222-3p, and miR-409-3p) are consistently downregulated in both. miR-106b-5p and miR-222-3p are currently under investigation in preclinical and clinical trials for their therapeutic potential in T1D. As therapeutic strategies, ungulated miRNAs may be targeted with inhibitors, while downregulated miRNAs can be restored using mimics.

A comprehensive analysis of T1D pathogenesis and its associated complications has identified a range of consistently dysregulated miRNAs. As summarized in Figure 3, miRNA expression patterns in T1D patients and animal models show considerable overlap. In T1D patients, 44 miRNAs are upregulated, while 22 are downregulated (Table 1). Similarly, T1D animal models exhibit 5 upregulated and 10 downregulated miRNAs (Table 2). Notably, miR-21, miR-29a, miR-142-3p/5p, miR-145, and miR-323-3p is consistently upregulated in both T1D patients and murine models. Conversely, miR-10b-5p, miR-16-5p, miR-17-5p, miR-106b-5p, miR-126-5p, miR-143-3p, miR-150, miR-216a, miR-222-3p, and miR-409-3p are consistently downregulated in both populations.

Additionally, miR-106b-5p and miR-222-3p have emerged as potential therapeutic targets due to their roles in pancreatic β-cell function. Tsukita et al. [180] found that bone marrow transplantation (BMT) restored pancreatic islets in STZ-induced diabetic mice while increasing miR-106b and miR-222 levels in serum exosomes and islets. Exosomal miRNA analysis showed elevated miR-106b-5p and miR-222-3p in the culture media of bone marrow cells from STZ-BMT mice. Notably, administering miR-106b-5p and miR-222-3p mimics enhanced β-cell proliferation and improved hyperglycemia by downregulating the Cip/Kip family, promoting β-cell regeneration [180]. A miR-216a mimic nanodrug has also been shown to enhance β-cell proliferation via PTEN inhibition, leading to increased insulin production [178]. Another study identified miR-142-3p as a key regulator of islet autoimmunity in NOD mice. Inhibiting miR-142-3p with an LNA-miRNA inhibitor enhanced regulatory T-cell stability by targeting TET2, reducing islet autoimmunity in diabetic mice [181].

Furthermore, miR-21 [29,42,43,44] and miR-146a [39,67,77,78,79,144,145]—both implicated in inflammatory and autoimmune responses—have emerged as potential therapeutic targets. Modulating these dysregulated miRNAs using inhibitors or mimics (Figure 3) may help reduce immune-mediated β-cell destruction and inflammation in T1D. Targeting these pathways could slow disease progression and potentially prevent its onset. As research advances, miRNA-based therapies may offer a novel approach to preserving β-cell function and improving T1D outcomes.

The sequences of most mature miRNA species are identical between mice and humans [26]. However, approximately 20% of miRNA sequences exhibit variations, potentially due to post-transcriptional modifications and/or PCR and sequencing errors [26]. miRNAs are highly conserved across animal species [182], and miRNA-mediated gene silencing mechanisms have been evolutionarily preserved [183]. According to miRBase (v22.1), mice and humans possess approximately 1234 and 1917 miRNA genes, respectively [184]. While miRNA homologs between the two species are strongly conserved [185], no comprehensive studies have analyzed overall sequence differences. Notably, the seed regions (nucleotides 2–8 at the 5′ end), which are critical for target recognition, are highly conserved among mammalian mRNAs’ 3′ untranslated regions [186]. Furthermore, 51 genes linked to T2D and obesity at miRNA-mRNA binding sites are conserved between mice and humans [187]. The commonly dysregulated miRNAs in T1D patients and murine models (Figure 3) also exhibit strong conservation, particularly in their seed regions (100% identical), suggesting that these miRNAs may regulate the same target genes in both species.

## 6. Conclusions and Future Study

T1D results from the loss of pancreatic β-cells through autoimmune or idiopathic processes, yet the precise molecular mechanisms driving β-cell destruction remain incompletely understood. Furthermore, no therapeutics have been developed to effectively reverse disease progression in T1D.

miRNA-based therapeutics offer a unique advantage, as a single miRNA can regulate the expression of multiple genes within related pathways, ranging from tens to hundreds [188]. These therapies operate through two primary strategies: miRNA mimics, which restore deficient miRNAs to reduce pathological gene expression, and miRNA inhibitors, which suppress overactive miRNAs to recover protein synthesis [14,179]. Additionally, because miRNAs are endogenous molecules, they exhibit low immunogenicity, reducing the likelihood of immune rejection [189].

Although no miRNA-based therapies have received FDA approval to date, numerous clinical trials, as reviewed by Zogg et al. [14], are exploring their therapeutic potential. These studies demonstrate the broad applicability of miRNA therapeutics in various diseases, including diabetes and autoimmune disorders [14]. This review highlights key preclinical studies investigating miRNA-based therapies in T1D, detailing their therapeutic efficacy, dysregulated miRNAs in T1D patients and murine models, and their associated pathways and target genes.

Despite their promise, miRNA therapeutics face several challenges. First, miRNA mimics and inhibitors are highly susceptible to degradation in circulation, limiting their stability and therapeutic efficacy. Second, intracellular delivery remains inefficient, as many miRNAs become sequestered in endosomes rather than reaching their target sites in the cytoplasm. Developing advanced delivery systems and enhancing endosomal escape mechanisms are crucial for improving gene silencing efficiency. Lastly, miRNAs can induce off-target effects by regulating multiple genes across different pathways and cell types, potentially leading to unintended gene silencing. Strategies such as sequence optimization, chemical modifications, and targeted delivery approaches can enhance specificity and minimize off-target effects, thereby improving the safety and efficacy of miRNA-based therapies.

Although current miRNA-based therapeutics face obstacles, rapidly advancing preclinical and clinical research, along with interdisciplinary innovations, may soon overcome these challenges. Given their remarkable therapeutic potential, miRNA-based treatments hold promises not only for T1D but for a broad spectrum of diseases.

## Data Availability

Not applicable.

## Abbreviations

T1D: type 1 diabetes; T2D, type 2 diabetes; GD, gestational diabetes; ER, endoplasmic reticulum; AAbs, autoantibodies; HLA, human leukocyte antigen; miRNAs, microRNA; mRNAs, messenger RNAs; PBMC, peripheral blood mononuclear cells; Treg, regulatory T-cell; NOD mice, non-obese diabetic mice; STZ, Streptozotocin; ROS, reactive oxygen species; BMT, bone marrow transplantation.
